# Altered immunity in crowded *Mythimna separata* is mediated by octopamine and dopamine

**DOI:** 10.1038/s41598-018-20711-8

**Published:** 2018-02-16

**Authors:** Hailong Kong, Chuanlei Dong, Zhen Tian, Nian Mao, Cheng Wang, Yunxia Cheng, Lei Zhang, Xingfu Jiang, Lizhi Luo

**Affiliations:** 1 College of Horticulture and Plant Protection, Yangzhou University, Wenhui East Road, NO. 48, Yangzhou, 225009 China; 2 State Key Laboratory for Biology of Plant Diseases and Insect Pests, Institute of Plant Protection, Chinese Academy of Agricultural Sciences, Yuanmingyuan West Road, No. 2, Beijing, 100193 China

## Abstract

Similar to pathogenic infection, high population density alters insects’ prophylactic immunity. Density-dependent prophylaxis has been reported in many polyphenic insects, but the regulatory mechanism underlying this phenomenon remains unclear. The biogenic monoamines are known to play critical roles in mediating insect immune responses. In the current study, the immune capacity and the levels of three biogenic monoamines were investigated in the polyphenic larvae of *Mythimna separata*, reared at the densities of 1, 2, 5, 10, and 30 larvae per 650-mL jar. Concomitant with the increased phenoloxidase (PO) activity and total haemocyte count in the larvae at high densities (5, 10, 30 larvae/jar), the octopamine level was also increased. In contrast, the dopamine level was decreased, and the 5-hydroxytryptamine level was not significantly affected. Injection of octopamine induced significant increases in the total haemocyte count and PO activity. Conversely, epinastine, a specific antagonist of octopamine, decreased the total haemocyte count and PO activity. Another octopamine antagonist, phentolamine, inhibited the activity of PO and lysozymes. In addition, injection of dopamine induced a significant increase in PO activity and decreased the total haemocyte count and lysozyme activity. These results suggested that both octopamine and dopamine mediate the increases in total haemocyte count and PO activity in the crowded larvae.

## Introduction

Insects infected with pathogens are ubiquitous in nature. It has been found that insects living in high-density populations have evolved adaptive prophylactic mechanisms to cope with pathogens^1^. Individuals experiencing crowded conditions are predicted to be more resistant to pathogens than those experiencing low-density conditions, through facultative allocation of physiological resources to defence mechanisms. This phenomenon, termed “density-dependent prophylaxis”^1^, has been observed in many species that display phase polyphenism, including *Spodoptera exempta*^2^, *Mythimna separata*^3^, *Spodoptera littoralis*^4^, *Locusta migratoria*^5^ and *Loxostege sticticalis*^6^. Insects can mount an immune response when challenged with an infection^7^. In fact, an increased immune response is also observed in insects under high-density conditions. Increasing phenoloxidase (PO) activity, total haemocyte counts and lysozyme activity had been observed in some species from high-density conditions^2,4–6^. The immune system plays an important role in insects’ prophylactic response to pathogens. However, the mechanism regulating the immune response underlying high-density prophylaxis is not very clear.

Connections between the immune and nervous systems are ubiquitous in animals^8^. The immune system requires information about the environment in order to respond adaptively^8^, and the nervous system integrates sensory information and sends that information to other systems, including the immune system^9^. Linking the nervous system and immune organs are the biogenic monoamines^10^. Three biogenic amines have been implicated in the regulation of animal immune function: dopamine (DA), octopamine (OA) and serotonin (5-hydroxytryptamine, 5-HT). In response to bacterial infection, OA and 5-HT enhanced both haemocytic phagocytosis and nodule formation in the beet armyworm *Spodoptera exigua*^11^. OA also increased the number of circulating haemocytes in *S*. *exigua*^12^. In addition, the OA level increased in the haemolymph during immune challenge^13^, and OA enhanced phagocytosis in cockroach haemocytes^14^. 5-HT mediates haemocyte phagocytosis through the receptors 5-HT1B and 5-HT2B^15^. The dopamine-dependent signalling system operates in haemocytes to mediate phagocytic functions^16^. N-β-alanyldopamine synthase was induced in the mealworm, *Tenebrio molitor*, and in the medfly, *Ceratitis capitata*, by infection with *Escherichia coli*^17^. Genes for biogenic amine receptors were also expressed in the immune system of the Texas field cricket, *Gryllus texensis*^18^. All these data seem to indicate that the action of biogenic amines is probably linked with the immune function of insects.

The oriental armyworm, *Mythimna separata* (Lepidoptera: Noctuidae), a major long-distance migratory insect pest of grain crops in China and other Asian countries, shows typical density-dependent phase polymorphism^19–21^. Larval colour polymorphism, survival rates, development rate, feeding behaviour of larvae, and flight capacity, fecundity and energetic reserves in adults are affected by larval density^19,22,23^. Mitsui and Kunimi^24^ (1988) found that larvae from gregarious (high density) phases were more resistant than solitary (low density) phase larvae when perorally inoculated with nuclear polyhedrosis virus (PsNPV). Our preliminary results also showed that the resistance to the entomopathogenic fungus *Beauveria bassiana* and the bacterium *Bacillus thuringiensis* of larvae raised at high density was higher than that of larvae raised at low density. Therefore, *M*. *separata* larvae showed density-dependent prophylaxis.

PO activity, total haemocyte counts and lysozyme activity have been reported to cause increased resistance to disease in phase species, such as *S*. *exempta*, *S*. *littoralis*, *L*. *migratoria*, *L*. *sticticalis*, under crowded conditions^2,4–6^. The levels of octopamine, dopamine and 5-hydroxytryptamine were significantly higher in crowded *Gryllus bimaculatus* than in isolated individuals^25^. It is assumed that under larval crowding stress, *M*. *separata* may produce increased levels of biogenic amines, subsequently leading to increased immunity. Accordingly, the purpose of the present study is to examine (1) the effect of larval density on the immune response and levels of biogenic amines in *M*. *separata* and (2) the immune responses of *M*. *separata* injected with biogenic amines and their antagonists.

## Results

### Immune and size assays

**Figure 1:**
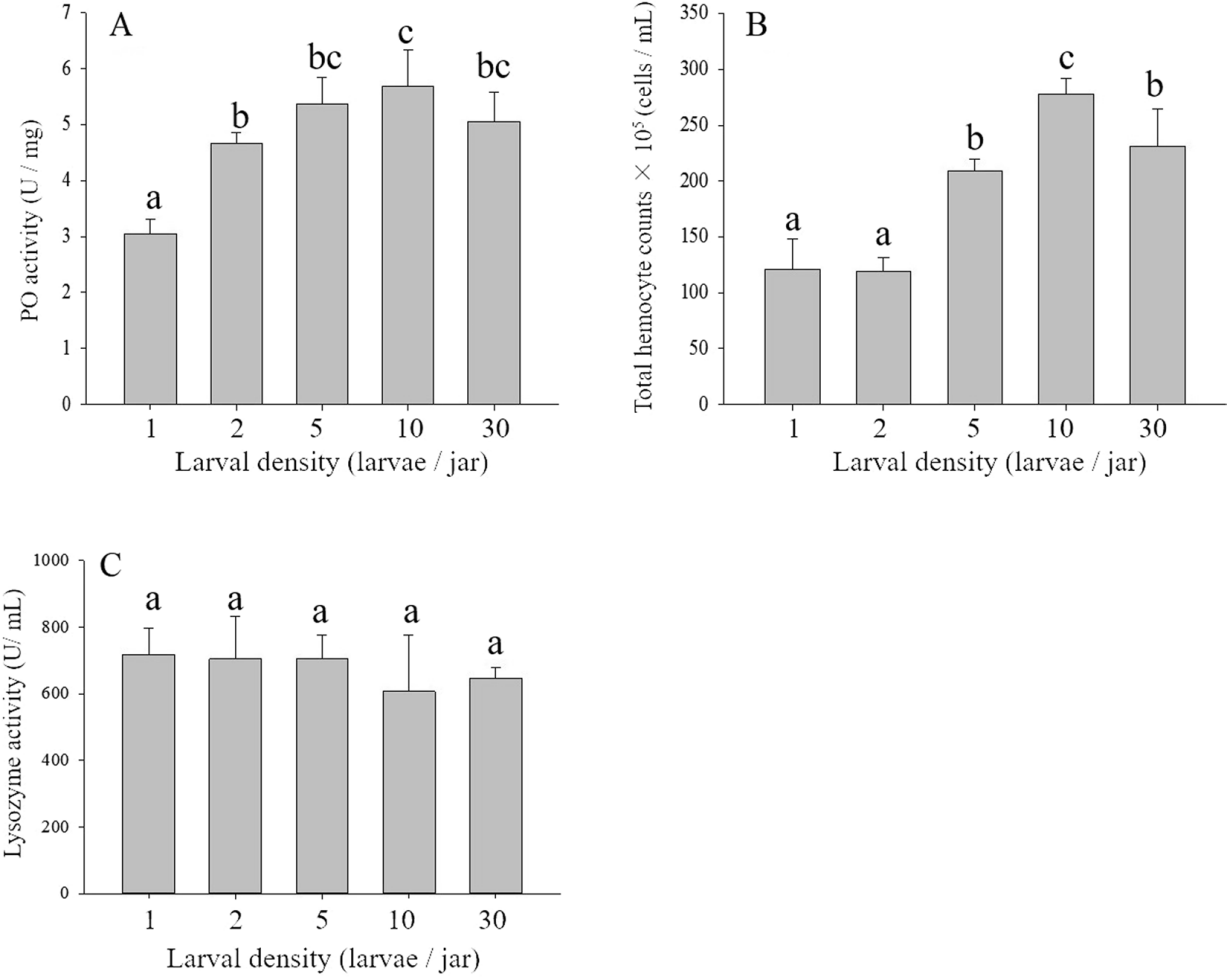
Effect of larval density on three measures of larval immune function in *M*. *separata*. (**A**) Phenoloxidase activity. (**B**) Total haemocyte count. (**C**) Lysozyme activity. Values are means ± SE. Different letters indicate significant differences among the densities of 1, 2, 5, 10 and 30 larvae/jar (P < 0.05).

Larval density significantly affected the PO activity (F = 14.92; df = 4; P < 0.01; Fig. 1A). PO activity was highest for larvae kept at 10 larvae/jar and was significantly higher than that for larvae kept at 1 or 2 larvae/jar. However, it declined as larval density increased, although there was no significant difference in the PO activity of larvae between densities of 10 and 30 larvae/jar. Total haemocyte count was also significantly affected by larval density (F = 42.39; df = 4; P < 0.01; Fig. 1B); total haemocyte count was significantly increased with increased larval density. Larvae from densities of 5, 10 and 30 larvae/jar had significantly higher total haemocyte counts than larvae from densities of 1 and 2 larvae/jar. Larval lysozyme activity was not significantly different among the five density treatments (F = 0.81; df = 4; P = 0.54; Fig. 1C).

Larval density significantly affected the weight of the larvae (F = 4.09; df = 34; P < 0.01; Table 1). Weights of larvae reared at 1 larva/jar were significantly greater than those from the other, higher densities tested. The body length of larvae did not differ significantly among the five density treatments (F = 0.49; df = 34; P = 0.74; Table 1). However, the width of larvae was significantly affected by larval density (F = 12.22; df = 34; P < 0.01; Table 1); the body width of larvae from the 1 larva/jar treatment was greatest and was significantly greater than those of larvae from the treatments with 2, 10 and 30 larvae/jar.

**Table 1:** Effects of larval density on the sizes and weights of *M*. *separata* larvae.

Density (larvae/jar)	Weight (g)	Length (mm)	Width (mm)
1	0.55 ± 0.06a	27.79 ± 1.55a	5.53 ± 0.27a
2	0.47 ± 0.06b	26.97 ± 2.63a	5.10 ± 0.37b
5	0.47 ± 0.07b	27.27 ± 1.09a	5.20 ± 0.26ab
10	0.45 ± 0.07b	27.39 ± 2.68a	4.96 ± 0.36b
30	0.40 ± 0.08b	26.03 ± 3.67a	4.39 ± 0.31c

Data are presented as the mean ± SE. Within a column, values followed by different letters are significantly different at the 5% level as determined by Duncan’s multiple range test.

### Biogenic amine assays

**Figure 2:**
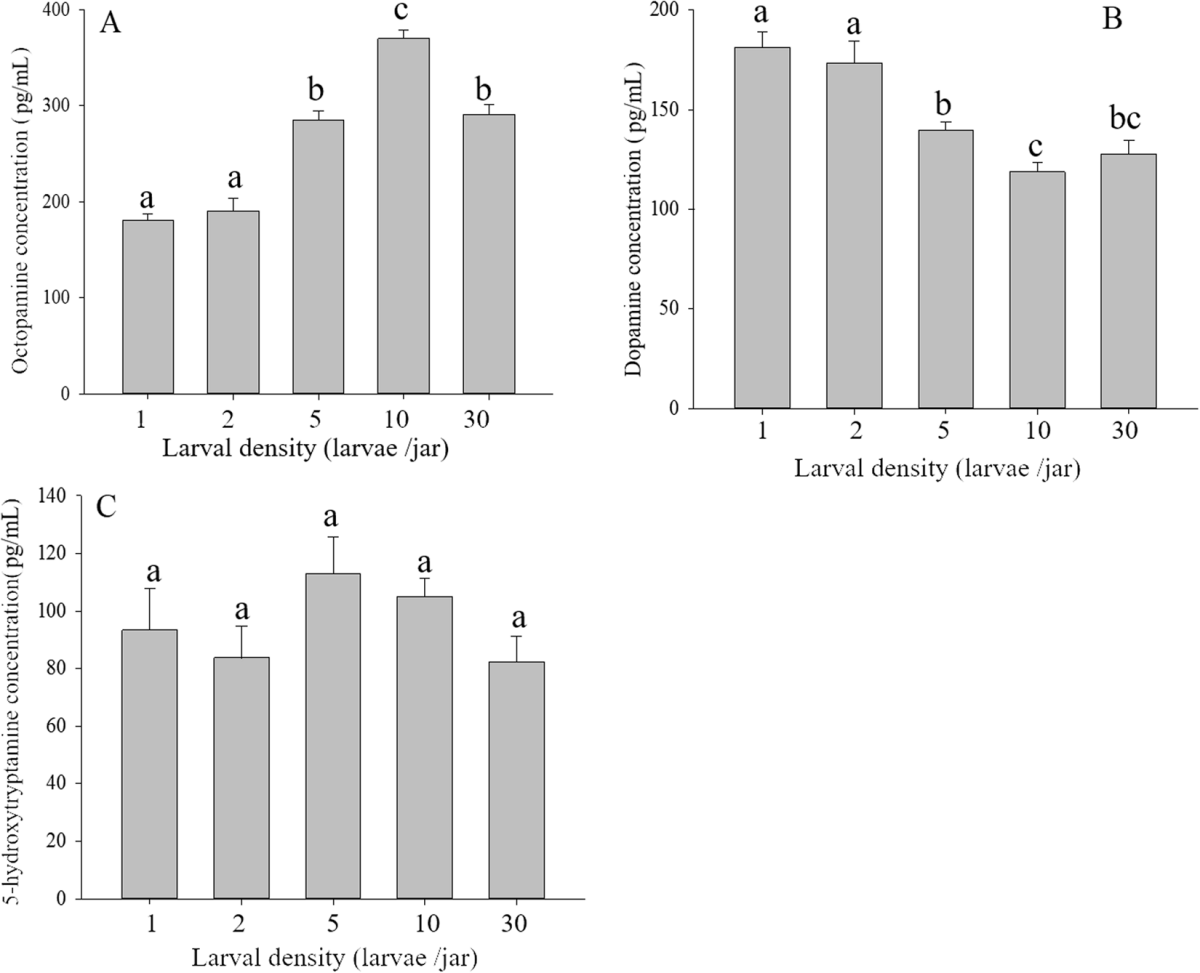
Effects of larval density on the levels of biogenic amines in larvae of *M*. *separata*. (**A**) Octopamine. (**B**) Dopamine. (**C**) 5-hydroxytryptamine. Values are means ± SE. Different letters indicate significant differences among the densities of 1, 2, 5, 10 and 30 larvae/jar (P < 0.05).

Larval density significantly affected the octopamine level in larval haemolymph (F = 189.70; df = 4; P < 0.01; Fig. 2A). The octopamine level was highest for larvae from the treatment with 10 larvae/jar, in which it was significantly higher than that of larvae from the other density treatments. However, the octopamine level declined as larval density increased. Larval density also had a significant influence on the dopamine level in larval haemolymph (F = 42.18; df = 4; P < 0.01; Fig. 2B). The dopamine level of larval haemolymph from the densities of 1 and 2 larvae/jar was significantly higher than that for larvae from the densities of 5, 10 and 30 larvae/jar. The level of 5-hydroxytryptamine did not differ significantly among the five density treatments (F = 2.30; df = 4; P = 0.13; Fig. 2C).

### Immune assays after injection with octopamine

**Figure 3:**
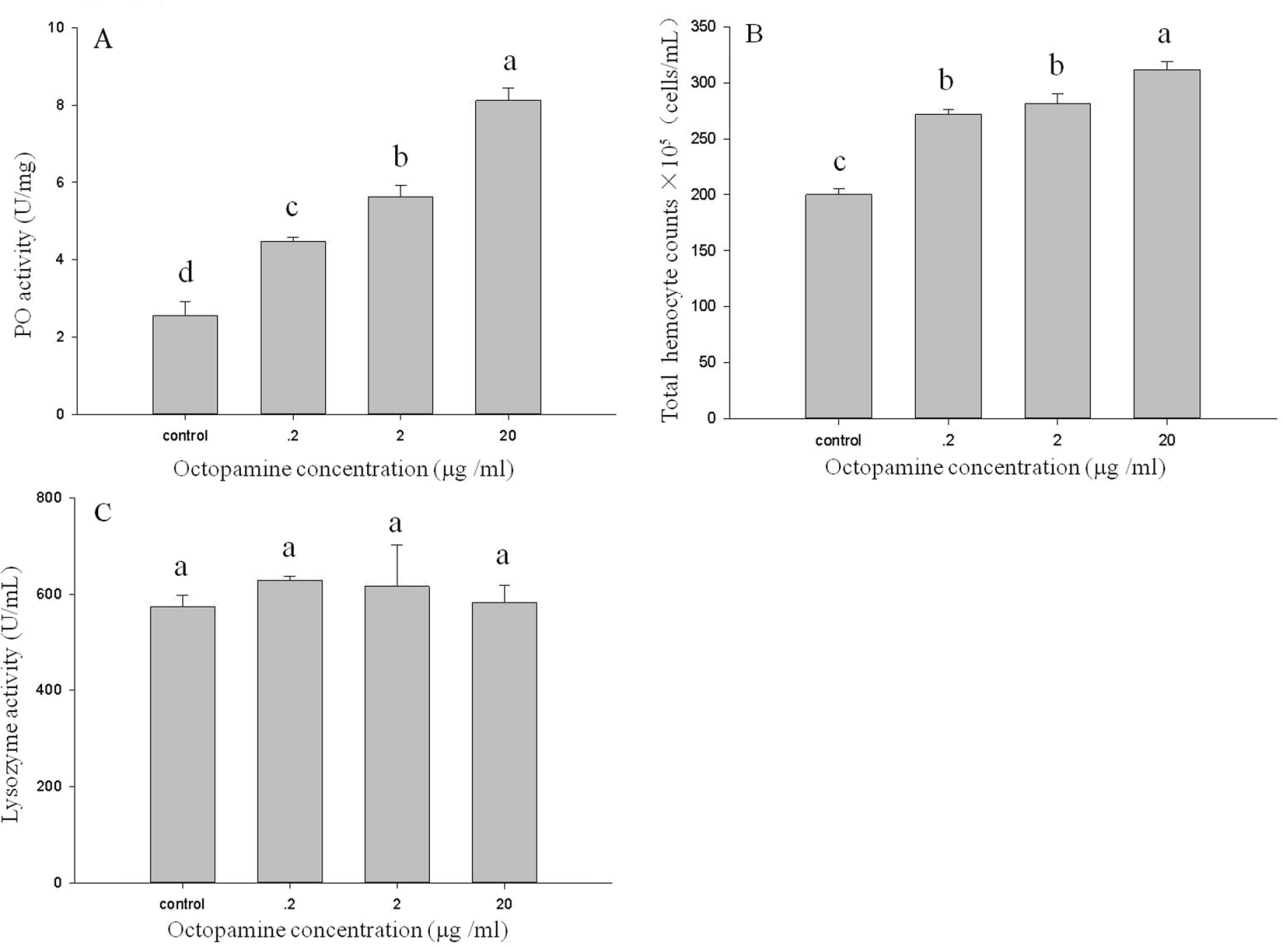
The three measures of immune function of *M*. *separata* larvae injected with different concentrations (0.2, 2, 20 µg/mL) of octopamine solution. (**A**) Phenoloxidase activity. (**B**) Total haemocyte count. (**C**) Lysozyme activity. Values are means ± SE. Different letters indicate significant differences at P < 0.05.

PO activity and total haemocyte count were significantly influenced by injection of octopamine solutions. PO activity increased significantly in larvae that received octopamine at 0.2, 2 and 20 µg/mL (F = 68.68; df = 3; P < 0.01; Fig. 1A). Similar results were also observed for the total haemocyte count (F = 48.86; df = 3; P < 0.01; Fig. 1B), with concentrations of OA up to 20 µg/mL. However, there was no significant difference in the lysozyme activity among larvae injected with different concentrations of octopamine solution (F = 0.30; df = 3; P = 0.82; Fig. 3C).

### Immune assays after injection with octopamine antagonists, epinastine and phentolamine

**Figure 4:**
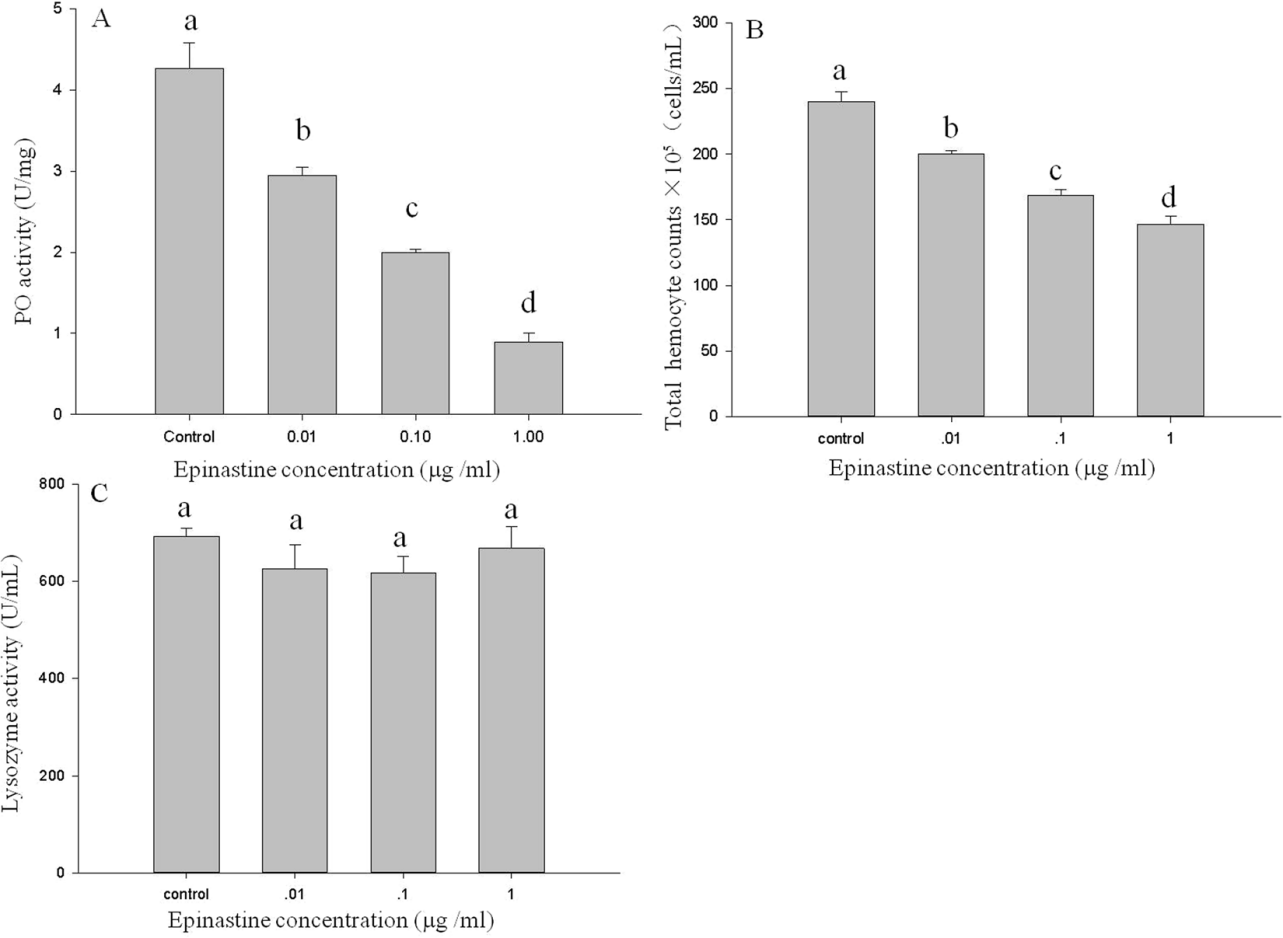
The three measures of immune function in *M*. *separata* larvae injected with different concentrations (0.01, 0.1, 1 µg/mL) of the octopamine antagonist epinastine. (**A**) Phenoloxidase activity. (**B**) Total haemocyte count. (**C**) Lysozyme activity. Values are means ± SE. Different letters indicate significant differences at P < 0.05.

**Figure 5:**
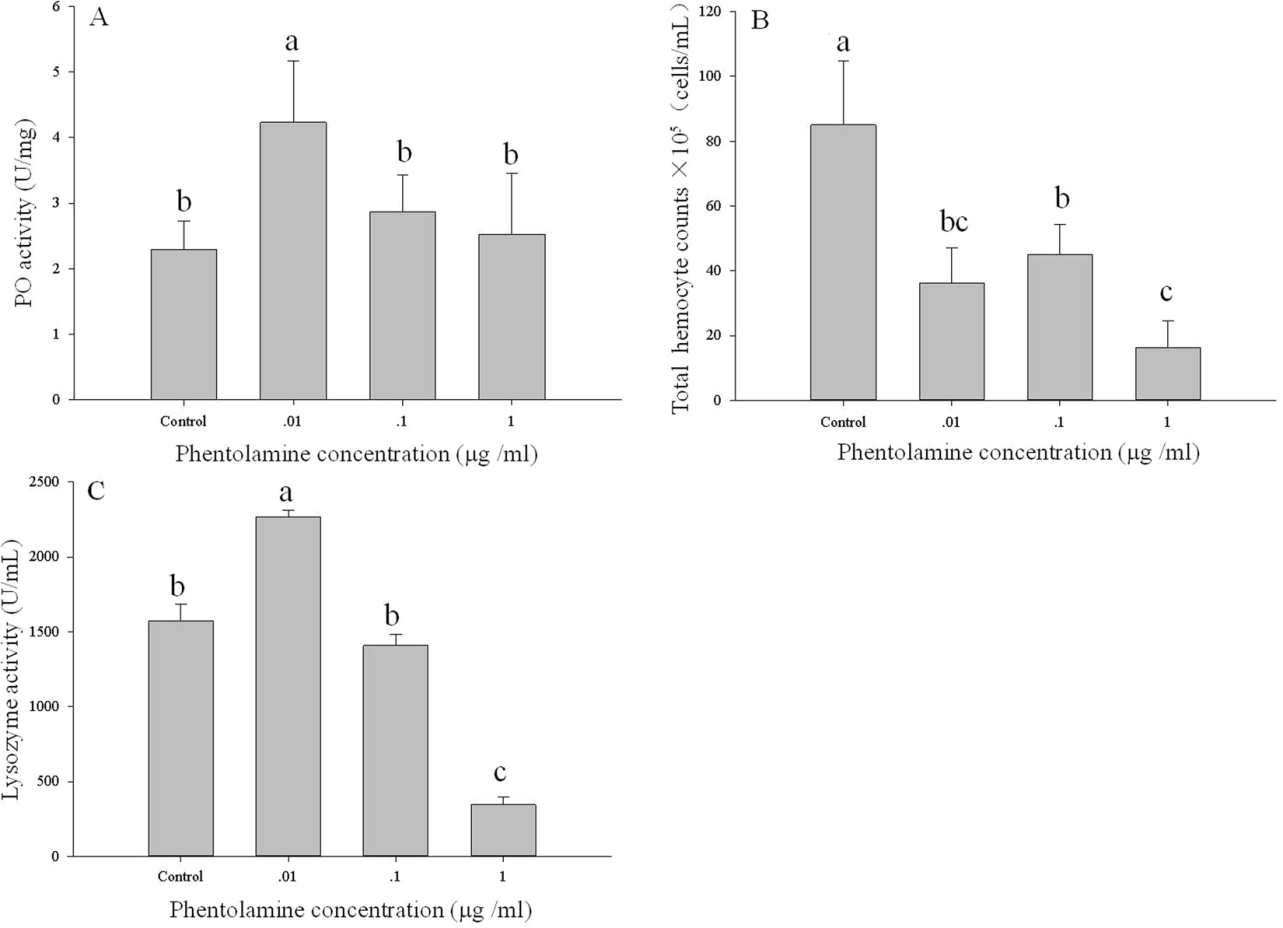
The three measures of immune function in *M*. *separata* larvae injected with different concentrations (0.01, 0.1, 1 µg/mL) of the octopamine antagonist phentolamine. (**A**) Phenoloxidase activity. (**B**) Total haemocyte count. (**C**) Lysozyme activity. Values are means ± SE. Different letters indicate significant differences at P < 0.05.

PO activity and total haemocyte count were also significantly influenced by injection solutions of the octopamine antagonists epinastine and phentolamine. A significant decrease in the PO activity was observed for larvae injected with epinastine at concentrations of 0.01, 0.1 and 1 µg/mL (F = 66.80; df = 3; P < 0.01; Fig. 4A). Similar results were also observed for the total haemocyte count (F = 53.90; df = 3; P < 0.01; Fig. 4B). However, lysozyme activity in the larvae was not significantly affected by epinastine (F = 0.88; df = 3; P = 0.49; Fig. 4C). Total haemocyte counts were also reduced in the larvae injected with phentolamine at concentrations of 0.01, 0.1 and 1 µg/mL (F = 15.18; df = 3; P < 0.01; Fig. 5B). In addition, different patterns were observed in the PO activity and lysozyme activity (F = 4.01; df = 3; P < 0.05; Fig. 5A; F = 207.64; df = 3; P < 0.01; Fig. 5C). Treatment with 0.01 µg/mL phentolamine significantly enhanced PO and lysozyme activity, but phentolamine at 1 µg/mL inhibited the lysozyme activity and did not significantly affect the PO activity.

### Immune assays after injection with dopamine

**Figure 6:**
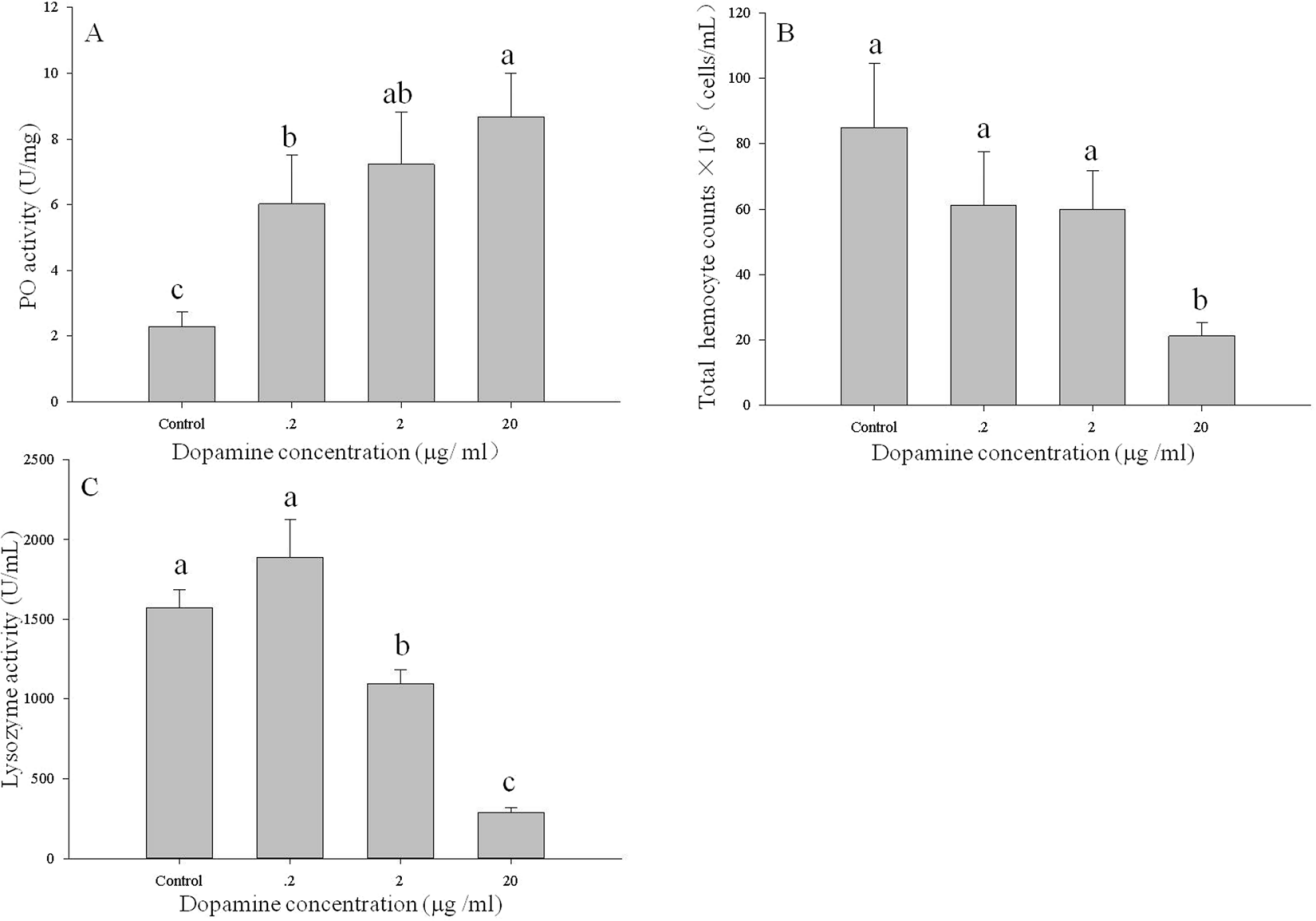
The three measures of immune function of *M*. *separata* larvae injected with different concentrations (0.2, 2, 20 µg/mL) of dopamine solution. (**A**) Phenoloxidase activity. (**B**) Total haemocyte count. (**C**) Lysozyme activity. Values are means ± SE. Different letters indicate significant differences at P < 0.05.

PO activity, total haemocyte count and lysozyme activity were significantly influenced by injection of dopamine solutions. Larvae that received dopamine at 0.2, 2 and 20 µg/mL showed significant increases in PO activity (F = 13.40; df = 3; P < 0.01; Fig. 6A). However, total haemocyte count and lysozyme activity were significantly decreased in larvae that received dopamine at 20 and at 2 or 20 µg/mL, respectively (F = 10.32; df = 3; P < 0.01; Fig. 6B. F = 48.35; df = 3; P < 0.01; Fig. 6C).

## Discussion

An insect mounts an immune response when challenged with an infection^7^. Research has demonstrated that increasing immune ability also occurred in larvae reared under high-density conditions, which show density-dependent prophylaxis. Wilson *et al*.^4^ found that PO activity in the haemolymph, cuticle and midgut of *S*. *littoralis* significantly increased with larval density. Resson *et al*.^2^ also found that haemolymph PO activity was significantly greater in crowded larvae than in solitary larvae of *S*. *exempta*. Wilson *et al*.^5^ found that total haemocyte count and lysozyme activity of *L*. *migratoria* were markedly higher with high population density (gregarious phase) than with low population density (solitary phase). Our results showed that PO activity and total haemocyte count of *M*. *separata* larvae from conditions of 5, 10, or 30 larvae/jar were significantly higher than those of larvae reared at 1 larva/jar. This suggests that the changes in immune parameters imposed by the larval density, such as increases in PO activity and total haemocyte counts, caused increases in the resistance of *M*. *separata* to disease.

It was noteworthy that although the PO activity of larvae reared at 10 larvae/jar was significantly higher than that of larvae reared at 2 larvae/jar, their weights were not significantly different. This situation is similar to that observed for total haemocyte counts. The weight of larvae reared in isolation was significantly greater than that of larvae reared at a density of 2 larvae/jar, but their total haemocyte counts were not significantly different. We therefore suggest that the variation in immune competence of larvae at different larval densities is not related to the size of the individual.

Most animals show a coordinated series of physiological changes in response to stress stimuli^26^. It has been shown that the levels of biogenic monoamines are altered in various insect species in response to unfavourable conditions. Our results showed that octopamine levels in the haemolymph of *M*. *separata* larvae reared at 5, 10 and 30 larvae/jar were significantly higher than those in larvae reared at 1 and 2 larvae/jar. Iba *et al*.^25^ found that the contents of octopamine in the corpora cardiaca were significantly higher in the crowded crickets than isolated ones. Octopamine was also elevated in the larvae of *Galleria mellonella* after bacterial infection^27^. The octopamine levels in the haemolymph of both the locust *Schistocerca americana gregaria* and the cockroach *Periplaneta americana* were significantly higher when exposed to mechanical, thermal and chemical stress than under normal conditions^28^. When worker honey bees (*Apis mellifera*) were fed pollen and exposed to CO_2_, brain dopamine levels were significantly lower than in untreated controls that were also fed pollen^29^. In contrast, we found that the dopamine levels after rearing at 5, 10 and 30 larvae/jar were significantly lower than after rearing at 1 or 2 larvae/jar. No significant difference in the dopamine level in the corpora cardiaca was observed between crowded and isolated crickets^25^. This inconsistent result may be due to different unfavourable conditions, different degrees of density stress, and different species (or regions of the body measured). On the other hand, the 5-HT level was not significantly affected by larval density. This is consistent with the results that the 5-HT level in the corpora cardiaca was not significantly different between crowded and isolated crickets^25^. Therefore, *M*. *separata* larvae may increase the octopamine level and decrease the dopamine level of haemolymph in response to high population density without changing the level of 5-HT.

The close correlation between the increasing octopamine level and the decreasing dopamine level may be related to the biosynthesis of biogenic amines. That is, in the process of octopamine biosynthesis, dopamine can be converted to tyramine by dopamine dehydroxylase, followed by β-hydroxylation of tyramine to form octopamine^30,31^.

Octopamine has been reported to mediate the cellular immune response in some insects^12,32^. Octopamine induced haemocyte phagocytosis and nodule formation in the cockroach *Periplaneta americana*^33^. Octopamine also increased the number of circulating haemocytes in *S*. *exigua*^12^. Similarly, octopamine accelerated the removal of bacteria from haemolymph in the greater wax moth, *Galleria mellonella*^27^. In response to bacterial infection, octopamine markedly enhanced both haemocytic phagocytosis and nodule formation in *Spodoptera exigua* larvae^11^. Our results showed that PO activity and total haemocyte counts were significantly stimulated by injection of various concentrations of octopamine solution. Epinastine is known as a highly specific antagonist of insect neuronal octopamine receptors^34^. When various concentrations of epinastine were injected into the haemolymph of *M*. *separata* larvae, significant decreases in the PO activity and total haemocyte counts in the larvae were observed. Treatments with phentolamine, another octopamine antagonist, at various concentrations (0.01, 0.1 and 1 µg/mL) and with a high concentration (1 µg/mL) of phentolamine significantly decreased total haemocyte counts and lysozyme activity, respectively. However, a low concentration (0.01 µg/mL) of phentolamine induced significant increases in PO activity and lysozyme activity. This may be because the phentolamine was recognized as foreign matter, leading to failure of its inhibitory effect. These results imply that octopamine may enhance haemocyte immune response and that octopamine also plays an important role in the larval resistance to infection. However, injection of octopamine can also result in a decline in resistance to bacteria. For example, the survival of crickets (*Gryllus texensis*) challenged with *Serratia marcescens* was decreased upon injection of octopamine^35^. Huang *et al*.^36^ also found that at a low concentration, octopamine stimulated haemocyte spreading and phagocytosis in the larvae of the striped stem borer, *Chilo suppressalis*, whereas at high concentrations, octopamine inhibited spreading and phagocytosis. Adamo^37^ also found that octopamine treatment decreased disease resistance in insects, despite the fact that it enhanced individual metrics of immune function. Therefore, the effects of octopamine on immune function may be related to the concentration of octopamine and to the insect species. Moreover, the effect of octopamine on immunity may be complex, and it requires further investigation.

Dopamine can also modulate immune functions^38^. Wu *et al*.^16^ reported that dopamine enhanced haemocyte phagocytosis via a D1-like receptor in the rice stem borer, *Chilo suppressalis*. Chang *et al*. (2006)^39^ found that dopamine depressed total haemocyte counts and PO activity in the tiger shrimp *Penaeus monodon*. Our findings showed that dopamine induced PO activity but decreased total haemocyte counts and lysozyme activity. These inconsistent results for immune parameters may be related to the tested time after injection and to the animal species.

Although the concentration of dopamine in larvae reared at higher densities (for example, 10 larvae/jar) is lower than that in larvae reared at lower densities (1 and 2 larvae/jar), the concentration of octopamine shows the opposite pattern. Moreover, among larvae reared at high densities, the content of octopamine exceeded that of dopamine. Therefore, compared with dopamine, octopamine plays a more dominant role in the modulation of the PO activity. Further, total haemocyte counts were increased by octopamine but decreased by dopamine. Due to the high content of octopamine and the low content of dopamine in larvae reared at higher densities, the total haemocyte counts were increased, and the inhibitory effect was blocked. However, the lysozyme activity of larvae reared at higher densities was not markedly affected by the lower dopamine contents.

In conclusion, the present study suggests that *M*. *separata* demonstrates density-dependent prophylaxis and that the octopamine and dopamine signalling pathways play important roles in immune modulation by adjusting PO activity and the total haemocyte count.

## Methods

### Colony maintenance

A laboratory colony of *Mythimna separata* was established with eggs supplied by the Institute of Plant Protection of the Chinese Academy of Agricultural Sciences, Beijing, China. Larvae were maintained at 23 ± 1 °C and under a 16 h light:8 h dark photoperiod until pupation and adult emergence. Adults emerging on the same day were collected and held together in a plastic cage with a 5-L capacity to allow mating and oviposition. Adults were provided daily with 10% (wt/vol) glucose solution as supplemental food. Eggs were laid on plastic cage with gauze until hatching. Larvae were maintained in plastic cages with a volume of 5 L and were fed daily with fresh leaves of maize (*Zea mays* L.). When larvae stopped feeding, sterilized soil containing 10% water was added to the cage to a depth of 5 cm to provide a substrate for cocoon formation, pupation, and adult emergence.

### Test subjects

The *M*. *separata* colony had been maintained for two generations when the experiments began. Newly hatched larvae were grouped and reared at five density treatments of 1, 2, 5, 10, or 30 larvae/jar (650-mL capacity, 10-cm diameter). The number of larvae for each density treatment was maintained throughout the feeding period. Excess food was provided by adding fresh leaves every morning. All insects were maintained at a constant temperature of 23 ± 1 °C with 70–75% relative humidity, and a 16 h light:8 h dark regime.

### Haemolymph sampling

On the second day of the fifth larval instar, larvae were cooled to torpor on ice and then sterilized by swabbing with 75% ethanol. An abdominal proleg was cut with a fine scalpel, and haemolymph was collected with a microcapillary pipette and transferred into a 1.5-mL centrifuge tube on ice to prevent melanization. Haemolymph samples were collected from groups of 30 larvae from each rearing density (1, 2, 5, 10, and 30 larvae/jar), and haemolymph from each rearing density were pooled as one biological replicate for the assays of PO and lysozyme activity and for haemocyte counts. Each biological replicate included three technical replicates. Three biological replicates were established for each density treatment. All the samples were then frozen at −80 °C until evaluation.

### Immune assays

#### Haemolymph PO activity

PO activity was analysed as described by Wilson *et al*. (2001)^5^. Briefly, 50 μL of haemolymph was added to 150 μL of phosphate buffer (pH = 7) in a plastic tube. Haemolymph PO activity was assayed spectrophotometrically by adding 1500 μL of 0.1 M L-dopa and 1000 μL of phosphate buffer (pH = 7) to 200 μL of haemolymph phosphate solution. Changes in the absorbance of the mixture within 3 min at 492 nm were measured using a time-dependent program and a lambda 25 UV/VIS spectrometer (Perkin Elmer Company, USA). Protein content was measured in the samples according to the method described by Bradford^40^. PO activity is expressed as PO units per milligram of protein, where 1 U was the amount of enzyme required to increase the absorbance by 0.001 min^−1^.

#### Total haemocyte count

Total haemocyte count was determined by adding 5 μl of haemolymph that had been stained with Giemsa for 5 min to the counting chambers of a haemocytometer. The haemocytes in the central square and four corners were counted under phase-contrast illumination and averaged to give an estimate of the number of haemocytes.

#### Lysozyme activity

Lysozyme activity was determined using a lysozyme kit (Nanjing Jiancheng Bioengineering Institute, Nanjing, China). First, 0.2 mL of haemolymph obtained as described above (see “Haemolymph sampling”) was mixed in a test tube with 2 mL of bacterial culture medium, and the two absorbance values at 530 nm were determined in a UV-2000 spectrophotometer (Unico Instrument Company of Shanghai, Shanghai, China) after 20 and 140 seconds, respectively. The lysozyme activity was determined using the two values according to the manufacturer’s instructions.

### Assays of body weights and size

On the second day of the fifth instar, the larvae from the density treatments of 1, 2, 5, 10 and 30 larvae/jar were weighed on an electronic balance (Sartorius, Germany), and the length and width of larvae from five density treatments were also assessed with callipers. For each density treatment, data from seven larvae were obtained.

### Assays of octopamine, dopamine and 5-HT

The octopamine, dopamine and 5-HT were determined based on the Insect Biogenic Amine ELISA Kit (Nanjing Jiancheng Bioengineering Institute, Nanjing, China). Standard curves were established based on standards of octopamine, dopamine and 5-HT, respectively. The haemolymph obtained from the procedure described above (10 μL), sample diluents (40 µL) and HRP-Conjugate reagent were combined and incubated for 60 min at 37 °C in microELISA wells. Each well was aspirated and washed with washing solution, followed by the addition of chromogen solution A (50 μL) and chromogen solution B (50 μL) and incubation for 15 min at 37 °C, after which 50 μL of stop solution was added. The absorbency at 450 nm was measured using a microplate reader (Power Wave XS2, Bio Tek Instrument Company of America, Ltd). The biological and technical replicates for the octopamine, dopamine and 5-HT assays were the same as described for the immunity assays.

### Effects of injected octopamine, octopamine antagonists (epinastine and phentolamine), or dopamine on the immune function

Octopamine, octopamine antagonists (epinastine and phentolamine) and dopamine were purchased from Sigma-Aldrich. Octopamine, epinastine, phentolamine and dopamine were dissolved in phosphate-buffered saline (PBS) and sterilized with 0.2-μm membrane filters. PBS was prepared at pH 7.4 with 50 mM sodium phosphate buffer and 0.7% NaCl. Various concentrations of octopamine (0.2, 2, 20 μg/μl), epinastine (0.01, 0.1, 1 μg/μl), phentolamine (0.01, 0.1, 1 μg/μl), and dopamine (0.2, 2, 20 μg/μl) solutions were injected into haemocoel through the abdominal proleg of fifth-instar larvae in a volume of 2 μL, using a 10-μL micro-syringe after surface sterilization with 70% ethanol. Injected larvae were placed in Petri dishes with leaves of corn under normal rearing conditions for 24 h.

Haemolymph samples were collected from groups of 30 larvae injected with octopamine, an octopamine antagonist (epinastine or phentolamine) or dopamine, as indicated. Haemolymph from larvae from each treatment were pooled as one biological replicate for the assays of PO and lysozyme activity and for haemocyte counts. Three biological replicates were established and used for three technical replicates. The haemolymph was collected and then frozen at −80 °C for the assays of phenoloxidase activity, total haemocyte count and lysozyme activity.

### Data analysis

All numeric values obtained from this study are presented as the mean ± SE. All data were assessed and showed a normal distribution using the Kolmogorov-Smirnov test. Differences among the different treatments were tested using one-way ANOVA followed by Duncan’s multiple range test. All statistical procedures were performed with SPSS 10.0.
